# Evaluation of the Applicability of Different Age Determination Methods for Estimating Age of the Endangered African Wild Dog (*Lycaon Pictus*)

**DOI:** 10.1371/journal.pone.0164676

**Published:** 2016-10-12

**Authors:** Moreangels M. Mbizah, Gerhard Steenkamp, Rosemary J. Groom

**Affiliations:** 1 Wildlife Conservation Research Unit, Department of Zoology, University of Oxford, Recanati-Kaplan Centre, Tubney, Oxford, United Kingdom; 2 Department of Companion Animal Clinical Studies, Faculty of Veterinary Science, University of Pretoria, Onderstepoort, South Africa; 3 Department of Zoology, University of Johannesburg, Auckland Park, South Africa; 4 African Wildlife Conservation Fund, Chishakwe Ranch, Birchenough Bridge, Zimbabwe; New York Institute of Technology, UNITED STATES

## Abstract

African wild dogs (*Lycaon pictus)* are endangered and their population continues to decline throughout their range. Given their conservation status, more research focused on their population dynamics, population growth and age specific mortality is needed and this requires reliable estimates of age and age of mortality. Various age determination methods from teeth and skull measurements have been applied in numerous studies and it is fundamental to test the validity of these methods and their applicability to different species. In this study we assessed the accuracy of estimating chronological age and age class of African wild dogs, from dental age measured by (i) counting cementum annuli (ii) pulp cavity/tooth width ratio, (iii) tooth wear (measured by tooth crown height) (iv) tooth wear (measured by tooth crown width/crown height ratio) (v) tooth weight and (vi) skull measurements (length, width and height). A sample of 29 African wild dog skulls, from opportunistically located carcasses was analysed. Linear and ordinal regression analysis was done to investigate the performance of each of the six age determination methods in predicting wild dog chronological age and age class. Counting cementum annuli was the most accurate method for estimating chronological age of wild dogs with a 79% predictive capacity, while pulp cavity/tooth width ratio was also a reliable method with a 68% predictive capacity. Counting cementum annuli and pulp cavity/tooth width ratio were again the most accurate methods for separating wild dogs into three age classes (6–24 months; 25–60 months and > 60 months), with a McFadden’s Pseudo-R^2^ of 0.705 and 0.412 respectively. The use of the cementum annuli method is recommended when estimating age of wild dogs since it is the most reliable method. However, its use is limited as it requires tooth extraction and shipping, is time consuming and expensive, and is not applicable to living individuals. Pulp cavity/tooth width ratio is a moderately reliable method for estimating both chronological age and age class. This method gives a balance between accuracy, cost and practicability, therefore it is recommended when precise age estimations are not paramount.

## Introduction

Age determination by means of teeth is one of the most practical and accurate methods for estimating the age of animals and animal remains. Age determination has become increasingly important to wildlife management and conservation efforts, because it significantly improves the quality of data on population structure, growth rates, onset of sexual maturity, fertility peak, age specific mortality rates, life span, as well as social behaviour [1–4]. Studying the age structure of a population can also provide hints about population growth and stability as well as ecological processes and their impact on population dynamics [5, 6]. Furthermore, studies of genetics, diseases, evolutionary ecology, life-history strategies and population dynamics require information on age-related parameters [7, 8]

African wild dog (*Lycaon pictus)* conservation programs would benefit considerably from being able to accurately assign age by both year and by age class. Yet information about the ages of individuals is often challenging to collect in the wild and effective monitoring of the animals can be difficult and costly [8]. Additionally, validation studies of different age determination methods are expensive to carry out and often only a few known age specimens are available [9], particularly for species or populations that are endangered and at risk of extinction such as the African wild dog (hereafter wild dog). For these species, detailed population information is critical for management decisions and wildlife conservation strategies [10].

There is a dearth of studies on age determination of African mammals as compared to mammals from America and Europe. Although there is an increasing interest in this subject, no single method has yet been demonstrated to be entirely reliable for African mammals. The majority of age determination studies in Africa have been on herbivores; Ugandan waterbuck (*Kobus defassa ugandae)* [11], zebra (*Equus quagga boehmi)* [12], West African buffalo (*Syncerus caffer brachyceros)* [13] and African elephant (*Loxodonta Africana)* [14]. Studies on age determination of carnivores are still lacking and the only studies published relate to leopard (*Panthera pardus)* [15], lion (*Panthera leo)* [16] and side striped jackal (*Canis adustus)* [8]. To our knowledge, to date, no studies have been conducted on aging of wild dogs from teeth.

Several age determination methods have been tested to determine their reliability in estimating the age of canids such as dingoes (*Canis lupus dingo)*, coyotes (*Canis latrans)*, wolves (*Canis lupus)* and foxes (*Vulpes vulpes)*. These include counting cementum annuli [9, 17–19], tooth wear [9, 19], cranial suture closure [19] and pulp cavity ratios [19–21].

The aim of the present study is to determine the feasibility of the commonly used age determination methods for estimating age of wild dogs. This study attempts to provide a method, or a combination of methods, for estimating wild dog age, which is precise enough for population dynamic studies, inexpensive, user-friendly and can be used both on live animals in the field and on skulls or teeth from dead animals. The criteria established should be applicable to other wild dog populations around Africa.

## Materials and Methods

Skulls of wild dogs were collected from the Savé Valley Conservancy (SVC) between 2008 and 2013. SVC is a privately owned area of approximately 3,490 km^2^ in the semi-arid southeast region of Zimbabwe. Full permission was given by the landowners to conduct this study in the area. The project is also fully supported by and works under permits from the Zimbabwe Parks and Wildlife Management Authority and the Research Council of Zimbabwe. The study area has abundant populations of wildlife including all indigenous large carnivores [22]. Wild dog skulls were collected when a wild dog carcass was found either opportunistically in the field or when tracking a collared individual that was found deceased. Sample collections were opportunistic; no animals were killed specifically for this study. No ethical clearance was needed for this study because no live animals were involved; skulls were only picked up from dead wild dogs found during monitoring activities in the field.

Wild dogs in this area form part of the largest remaining population of this endangered species in the country and have been monitored nearly continuously since 1996 [23]. From 2005, the African Wildlife Conservation Fund (AWCF) has closely monitored the population. Since all individual wild dogs are known by unique colour markings on their pelage, AWCF knows the vast majority of the wild dogs in the SVC individually, and for many, their exact age is known. As part of their conservation efforts, the AWCF team recorded all wild dog mortalities and wherever possible collected the skulls for this analysis. If the skulls had no more soft tissue covering, they were not processed further. Where the skulls were fresher and still had soft tissue attached, they were buried for a few months for cleaning by natural soil organisms. Boiling was found to loosen the teeth, and sometimes break fragile skulls, so this method was avoided. Teeth were extracted by either carefully part-boiling the skull then working the tooth loose with pliers or by cutting the socket with a small hacksaw until the tooth loosened enough to allow removal with pliers.

Mandibular and maxillary left canine teeth were extracted from 29 wild dog skulls; however only the maxillary canines were used for all the age determination analysis in this study. The sex and age was recorded for each individual when available. For this study we divided the wild dogs into 4 age classes:

0–6 months (the age when they are pups and have deciduous dentition)
6–24 months (the age when they are young and mostly staying with the pack)
25–60 months (the age when they are dispersing and reproducing)
> 60 months (old age).

The 0–6 months age class was removed from all the age determination analysis because they have deciduous dentition, which are anatomically very different from the permanent dentition. Teeth with complicated crown fractures (fractures of the crown where the pulp cavity was exposed) were excluded from all the other age determination analysis except for counting cementum annuli.

### An overview of the age determination methods

Cementum is deposited in a biphasic manner and therefore annual rings form and this has been found to be very reliable in age determination studies. The challenge of this method is that you need to have an extracted tooth to perform the procedure. Different types of teeth have been used for cementum line counts however, canines are preferable since cementum deposition could be followed over a longer section and successive cementum lines are less narrowly spaced and less inclined to merge [16]. Counting cementum annuli remains the only method available for estimating the age of older individuals more precisely [16]. Measuring the pulp cavity/tooth width ratio is one of the most common methods used in age determination. At eruption the pulp cavity of the tooth is very wide as only primary dentine is present. During the remainder of the tooth’s life secondary dentine is laid down circumferential around the pulp and therefore the pulp chamber decreases in size [16]. This reduction in size can also be affected by diet, in order to standardise the use of width measurements in this species we decided to work on a ratio in order to minimise variation between individuals as well as sexes.

It is accepted that a tooth will undergo wear (attrition and or abrasion) throughout its life and this will remove enamel and dentine from the crown and thereby shortening the tooth over time that is decreasing tooth crown height. In theory older wild dogs should have shorter teeth. Tooth wear (measured by tooth crown width/crown height ratio) just like the pulp cavity/tooth width ratio helps us to standardise for a species by removing inter individual and inter sex variability. Age determination by measuring tooth weight is one of the less commonly used methods. With increasing age (while the tooth is still viable) cementum and dentine is deposited. This will increase the weight of the tooth. This is not thought to be linear as dentine and enamel will be removed by wear. Skull measurements in certain species are dimorphic and can help with the identification of sexes. As an individual matures and the skull sutures close the growth rate of the skull will slow down and a growth curve can be plotted.

### Pulp Cavity/Tooth Width Ratio

**Fig 1:**
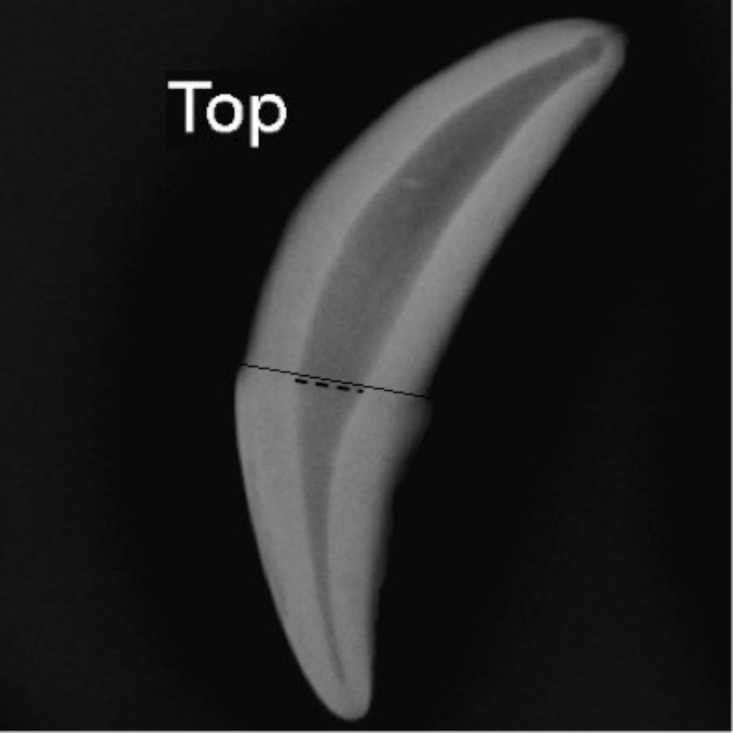
Radiograph of the African wild dog left maxillary (top) canine. All standardized measurements of the canine were performed by drawing a line on each radiograph at the cemento-enamel junction of the tooth. Pulp cavity width (dashed line) and tooth width (solid line) were then measured to calculate the pulp cavity/tooth width ratio.

One set of left side canine teeth (maxillary and mandibular canines) was radiographed on the same cassette. Both canine teeth from the same animal were placed with their lingual or palatal aspect centrally on the cassette and approximately 5 to 10 millimetres apart. Only two teeth were radiographed per cassette in order to reduce any image distortion that may take place. Images were made using a Siemens Polydoris 100 (Siemens PTY LTD, 300 Janadel Avenue, Halfway House, Midrand, South Africa) x-ray machine and a Fuji computed radiography (CR) system. For maximum detail a Fuji ID D 24X30 cm mammogram cassette (AXIM PTY LTD, 121 Gazelle Avenue, Corporate Park South, Midrand, South Africa) was used. Images were created using 44 kilovolts (kV) and 6 milli-amperes per second (mAs) and a film focal distance of 100 cm. The radiographic images were converted into JPEG files and processed using the Adobe Photoshop CC 2015 image software (Adobe Systems, USA). Standardized measurements of the left maxillary canine teeth were made by drawing a line on each radiograph at the cemento-enamel junction of the tooth (Fig 1). Tooth width and pulp cavity width were then measured at the drawn line using a ruler tool in Photoshop.

### Tooth Crown Height

**Fig 2:**
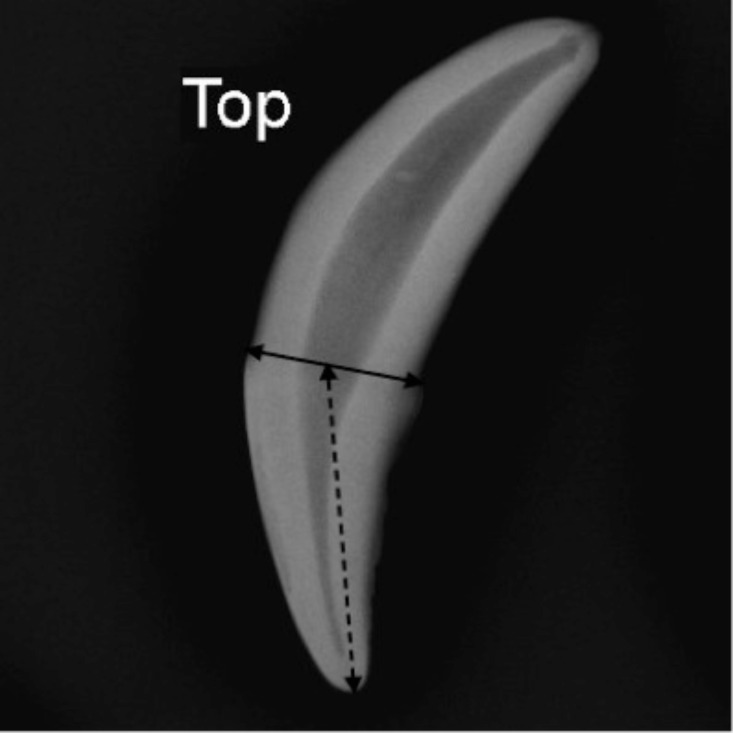
Radiograph of the African wild dog left maxillary (top) canine. The tooth crown height (dashed line) measured from the cemento-enamel junction to the crown tip and the crown width (solid line) was obtained by measuring the maximum distance between the mesial and distal contact points of the tooth at the cemento-enamel junction.

The JPEG files of the radiographic images obtained from the pulp cavity/tooth width ratio calculations above were used for this analysis. The images were processed using the Adobe Photoshop CC 2015 image software (Adobe Systems, USA). Tooth wear was assessed by the degree to which the canines had worn out as indicated by the distance from the cemento-enamel junction to the crown tip (tooth crown height) [16]. Standardized measurements of the canine teeth were made by drawing a line on each radiograph at the cemento-enamel junction of the tooth (Fig 2). A line was also drawn from the center of the cemento-enamel junction to the crown tip (Fig 2). Tooth crown height was measured from the cemento-enamel junction to the crown tip at the drawn line using a ruler tool in Photoshop.

### Tooth Crown Width/Crown Height Ratio

The JPEG files of the radiographic images obtained from the tooth crown height measurements were used in this analysis. As the tooth wears, crown height decreases, whereas crown width stays at least the same. The ratio of tooth crown width over tooth crown height increases with tooth wear and thus is a good measure of age [24]. The crown width was obtained by measuring the maximum distance between the mesial and distal contact points of the tooth on a line parallel to the occlusal plane at the cemento-enamel junction (Fig 2). The tooth crown height was measured from the cemento-enamel junction to the crown tip (Fig 2).

### Tooth Weight

Every whole (unbroken) left maxillary canine tooth was weighed on a Radwag AS X2 Analytical Balance (Lasec, 52 Old Mill Road, Ndabeni, Cape Town, South Africa) and the weight recorded to the nearest 0.001grams.

### Counting Cementum Annuli

Once all other analyses had been completed, the wild dog maxillary canines were sent to Matson’s Laboratory, Manhattan, MT, USA, for histological sectioning and staining. The laboratory, which has over 40 year’s experience in cementum analysis, examined each tooth without prior knowledge of its age. Matson’s Laboratory used 13 steps in processing teeth, as described on their website http://www.matsonslab.com and as described by Boertje, Ellis [25]. The teeth were processed by cleaning in a hot water bath, wiping with a nylon mesh, decalcifying in a weak acid solution, rinsing in water, dehydrating in isopropyl alcohol, clearing in toluene, and embedding in melted paraffin. The embedded teeth were then sectioned at a 14-micron thickness using a Leica RM 2100 Series rotary microtome (Leica, Buffalo Grove, IL).

**Fig 3:**
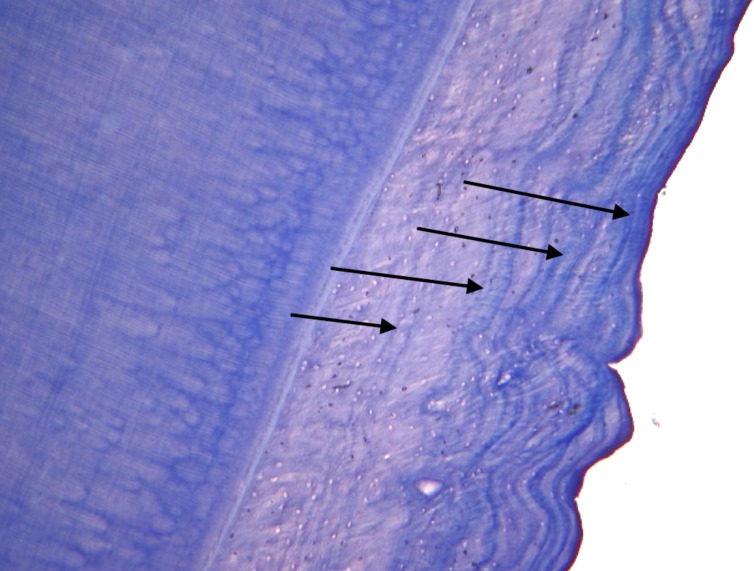
Photograph of sectioned canine at 60x magnification. Tooth belonging to African wild dog DWD058, date of death was 11 July 2013 and known age 4 years. Photograph taken at Matson’s Laboratory, Manhattan, MT, USA, which assigned the correct age of 4 years, with confidence (certainly level A), based on counting 4 cementum annuli (indicated by arrows).

**Fig 4:**
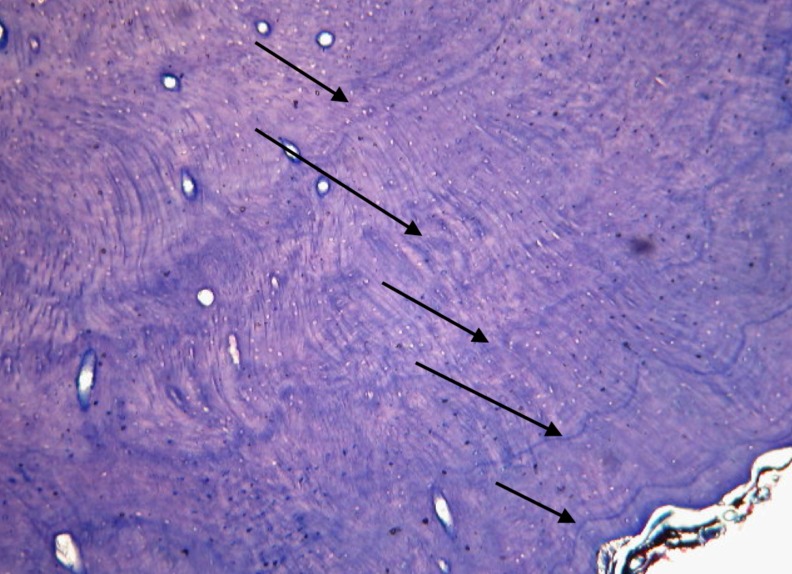
Photograph of sectioned canine at 60x magnification. Tooth belonging to African wild dog DWD057, date of death was 19 July 2013 and known age 5 years, Photograph taken at Matson’s Laboratory, Manhattan, MT, USA, which assigned the correct age of 5 years, with confidence (certainly level A), based on counting 5 cementum annuli (indicated by arrows).

The sections were then mounted on microscope slides, stained with Giemsa blood stain (Ricca Chemical Company, Arlington, TX), and a cover glass applied using Hypermount resin (Shandon, Inc., Pittsburgh, PA). The preparation obtained by this type of process is a “histological grade” preparation, enabling the microscopist to see all histological details in the material, Figs 3 and 4. The stained sections were examined at magnifications of 60 to 160X using Koehler illumination in a Leitz transmitted light microscope (Leica, Buffalo Grove, IL) equipped with plano objectives. An age estimate was received for each specimen together with age ranges for some of the specimens. An alphabetical certainty level for each specimen was also reported: A. result likely correct, Figs 3 and 4 and B. evidence less strong and error possible.

### Skull Measurements

**Fig 5:**
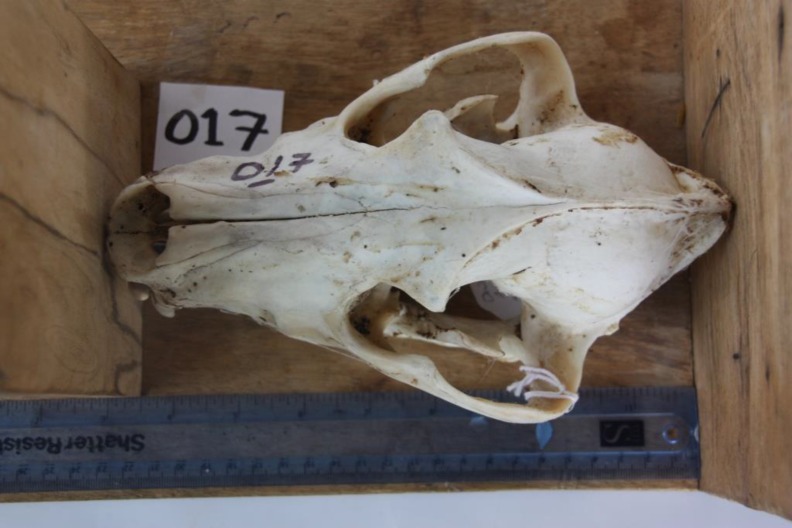
Skull length. African wild dog greatest skull length measured from the front of premaxilla to the back of the occiput.

**Fig 6:**
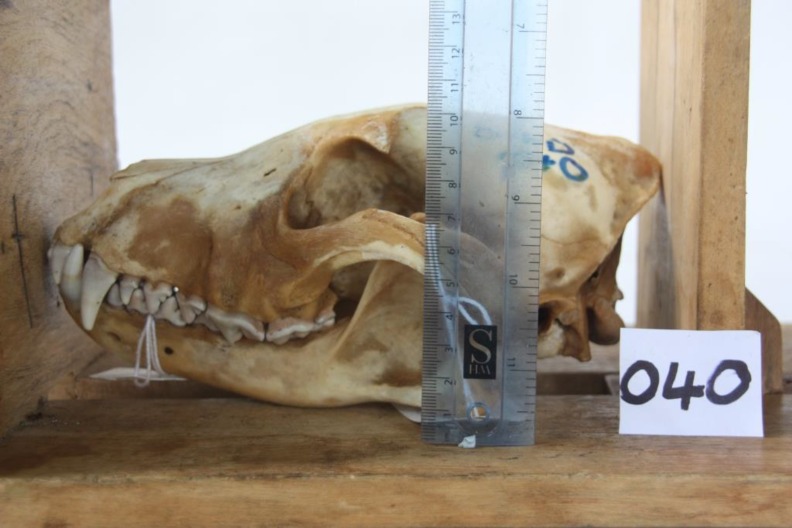
Skull height. African wild dog total skull height measured from the ventral border of the mandibles to the maximum height of the skull in between the frontal sinuses.

Standard skull measurements were taken on each cleaned wild dog skull using a ruler and keeping the skull between two wooden boards. The following three measurements were taken:

1. Skull length—The greatest skull length was measured from the front of premaxilla to the back of the occiput (Fig 5).
2. Skull width—The greatest width was measured across the zygomatic arches.
3. Skull height–The total skull height was measured from the ventral border of the mandibles to the maximum height of the skull in between the frontal sinuses (Fig 6).

### Statistical Analysis

In this study, 29 wild dog skulls and 29 maxillary canine teeth were examined to assess the viability of: (i) cementum annuli counts, (ii) pulp cavity/tooth width ratio, (iii) tooth wear (measured by tooth crown height) (iv) tooth wear (measured by tooth crown width/crown height ratio) (v) tooth weight and (vi) skull measurements (skull length, width and height) in estimating the chronological age and the age class of wild dogs. We first tested the interaction between the six age determination methods and wild dog sex for all chronological age and age class models. The interaction term had no significant influence on the response variables. Consequently we analysed both male and female wild dogs together.

Linear regression was done in the statistical package R [26] to calculate the performance of each of the six age determination methods in predicting wild dog chronological age. Regression diagnostics were first performed on each regression model to evaluate the model assumptions. The relationship between chronological age and pulp cavity/tooth width ratio was found to be non-linear. This relationship was close to exponential therefore log transformation was performed on both the response and predictor variables. Nonetheless the other five age determination models met the assumptions of regression and original data was used.

A statistical significance of 0.05 was considered and an F-statistics and a coefficient of determination (adjusted R^2^) as a measure the goodness of fit of the regression were reported. We further carried out a model selection to select a set of models that best described chronological age. Model selection was done using the corrected Akaike Information Criterion (AICc) adjusting for small sample size [27]. We considered the model with the lowest AICc score and highest AICc weight to be the most parsimonious and thus the “best” model explaining the variation in accuracy [27]. Ordinal logistic regression was done to evaluate the performance of the six age determination methods in predicting wild dog age class. The function ‘clm’ in the ‘ordinal’ package in R software was used to fit a cumulative link model using the logit link [26]. A goodness of fit using McFadden’s Pseudo-R^2^ was calculated for each model to measure overall model fit [28].

## Results

The sex was known for 24 of the wild dogs, 10 females and 14 males. The exact age of 17 individuals was known, based on known birth dates (within a month) and known dates of death (within about 2 weeks) (S1 Fig.).

### 1. Relationship between wild dog chronological age and the different age determination methods

#### Counting cementum annuli

**Fig 7:**
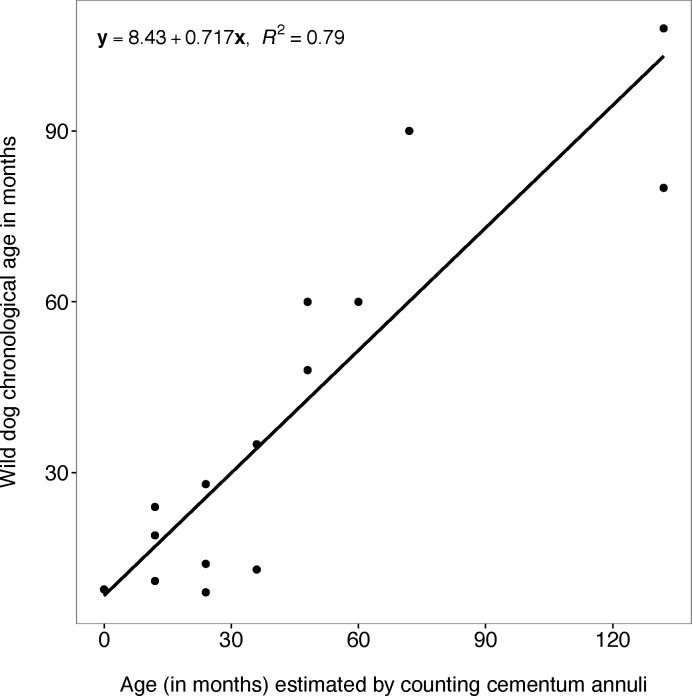
Chronological age and age estimated by counting cementum annuli. Plot showing the relationship between African wild dog chronological age and age estimated by counting cementum annuli of African wild dog left maxillary canines.

There was a strong relationship between wild dog chronological age and age estimated by counting cementum annuli in the linear regression model (F_1, 13_ = 54.56; P < 0.001) and the regression line approximated the real data points very well (y = 8.426 + 0.717 x; R^2^ = 0.793; 95% CI = 0.508 to 0.927; y: wild dog chronological age; x: age estimated by counting cementum annuli) (Fig 7).

#### Pulp cavity/tooth width ratio

**Fig 8:**
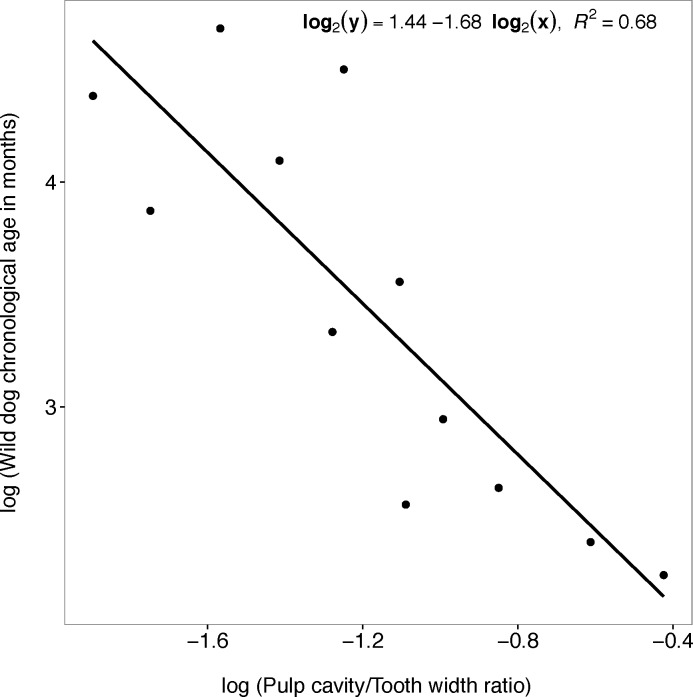
Chronological age and pulp cavity/tooth width ratio. Plot showing the relationship between African wild dog chronological age and the pulp cavity/tooth width ratio of African wild dog left maxillary canines.

There was a strong relationship between wild dog chronological age and pulp cavity/tooth width ratio in the linear regression model (F_1, 10_ = 24.63; P < 0.001). A one percentage increase in pulp cavity/tooth width ratio results in a 1.68 percentage decrease in chronological age and the regression line approximated the real data points well (log_2_ (y) = 1.445–1.679 log_2_ (x); R^2^ = 0.682; 95% CI = -2.432 to -0.925; y: wild dog chronological age; x: pulp cavity/tooth width ratio) (Fig 8).

#### Tooth wear (measured by tooth crown height)

**Fig 9:**
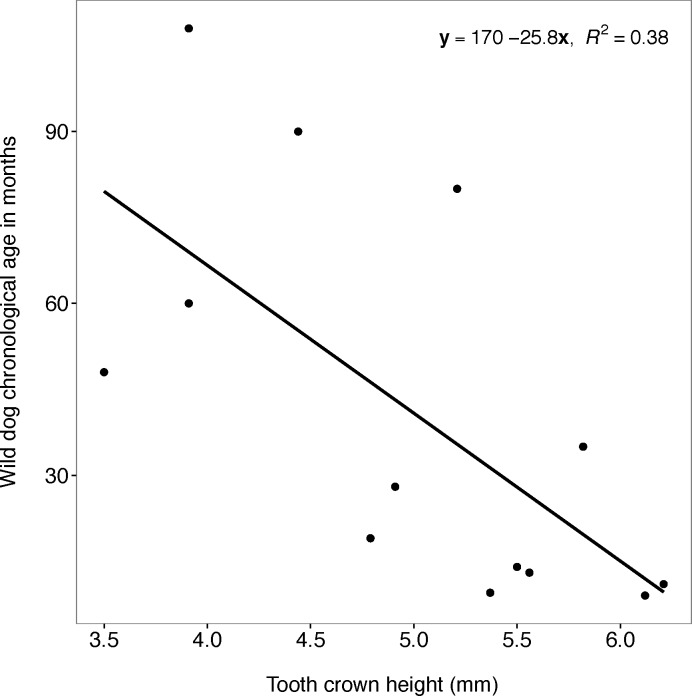
Chronological age and tooth crown height. Plot showing the relationship between African wild dog chronological age and tooth crown height of African wild dog left maxillary canines.

The relationship between wild dog chronological age and tooth crown height was significant in the linear regression model (F_1, 11_ = 8.505; P = 0.014) however, the regression line approximated the real data points poorly (y = 169.879–25.807x; R^2^ = 0.385; 95% CI = -45.284 to -6.331; y: wild dog chronological age; x: tooth crown height) (Fig 9).

#### Tooth wear (measured by tooth crown width/crown height ratio)

**Fig 10:**
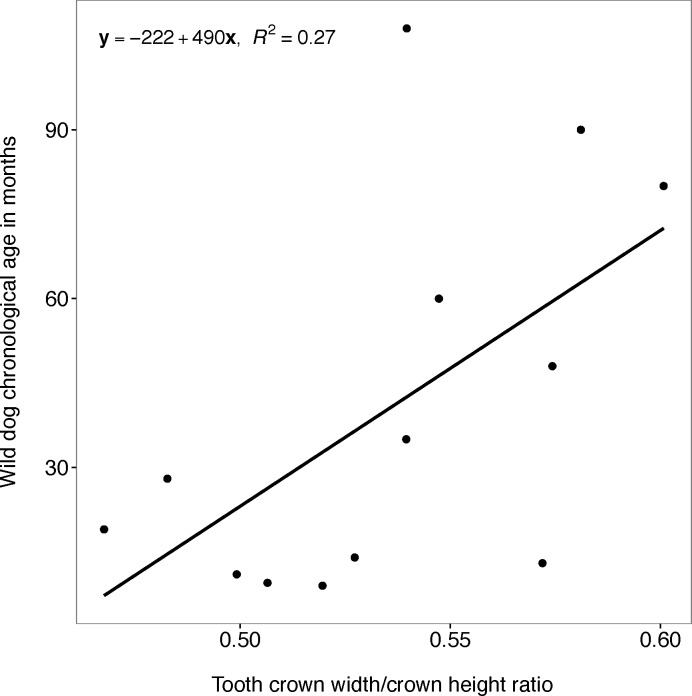
Chronological age and tooth crown width/crown height ratio. Plot showing the relationship between African wild dog chronological age and tooth crown width/crown height ratio of African wild dog left maxillary canines.

The relationship between wild dog chronological age and tooth crown width/crown height ratio was significant in the linear regression model (F_1, 11_ = 5.449; P = 0.04) but the regression line poorly approximated the real data points (y = -222 + 490x; R^2^ = 0.271; 95% CI = 27 to 952; y: wild dog chronological age; x: tooth crown width/crown height ratio) (Fig 10).

#### Tooth weight

The relationship between wild dog chronological age and tooth weight was not significant in the linear regression model (F_1, 11_ = 0.428; P = 0.5265).

#### Skull measurements

The relationship between wild dog chronological age and skull measurements was not significant for all measurements: skull length (F_1, 7_ = 1.057; P = 0.338), skull width (F_1, 7_ = 0.864; P = 0.384) and skull height (F_1, 7_ = 0.002; P = 0.962).

#### Model selection

The model with a combination of predictor variables counting cementum annuli and tooth crown height was chosen as the best model (F_1, 10_ = 43.46; P < 0.001, y = 77.115 + 0.607x - 12.906x_2_; R^2^ = 0.876; x: age estimated by counting cementum annuli; x_2:_ tooth crown height; y: wild dog chronological age). This model had the lowest AICc value, a delta AICc value of zero and the highest AICc weight (0.793) (Table 1). The model with the predictor variable counting cementum annuli was also a good model with a delta AICc < 5 (Table 1).

**Table 1:** Model selection ranked by Akaike’s information criterion (AIC) with small sample bias adjustment, AICc.

Variables	Intercept	TCH	Cementum	df	logLik	AICc	delta AICc	weight
Cementum + TCH	77.12	-12.91	0.607	4	-49.03	111.1	0	0.793
Cementum	6.978		0.723	3	-53.05	114.8	3.7	0.125

Results are for a subset of models with delta AICc < 5. The model with counting cementum annuli (Cementum) and tooth crown height (TCH) was the best model.

### 2. Relationship Between Wild Dog Age Class And The Different Age Determination Methods

#### Counting cementum annuli

A unit increase in age estimated by counting cementum annuli was significantly associated with a decrease in the log odds of being in the 6–24 months age class, with a log odds ratio of -0.20 (95% CI = -0.474 to -0.067), P = 0.038, McFadden’s Pseudo-R^2^ = 0.705. The probability calculated from threshold coefficients showed that the most likely age category of age estimated by counting cementum annuli of below 30 months is the 6–24 months age class. The most likely age category of age estimated by counting cementum annuli of between 30 months and 62 months is the 25–60 months age class. The most likely age category of age estimated by counting cementum annuli of above 62 months is the > 60 months age class.

#### Pulp cavity/tooth width ratio

**Fig 11:**
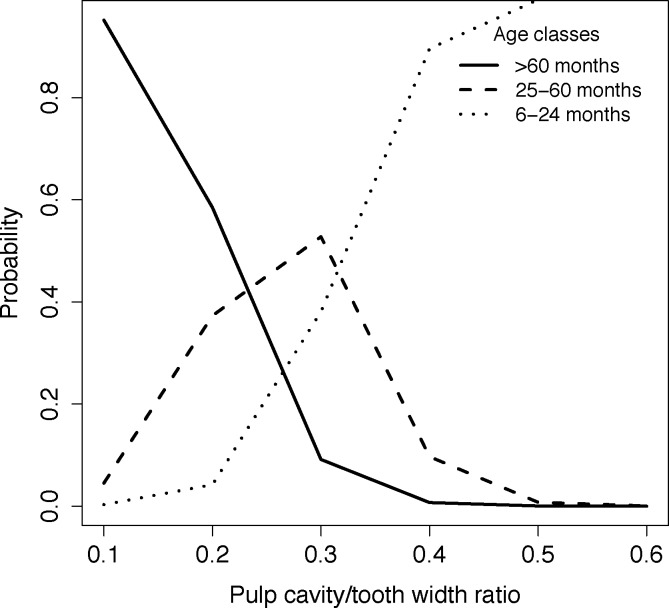
Age class and pulp cavity/tooth width ratio. Plot showing the probability of each African wild dog age class (6–24 months, 25–60 months and > 60 months) in relation to the pulp cavity/tooth width ratio of African wild dog left maxillary canines.

A unit increase in pulp cavity/tooth width ratio was significantly associated with an increase in the log odds of being in the 6–24 months age class, with a log odds ratio of 26.38 (95% CI = 8.095 to 56.542), P = 0.027, McFadden’s Pseudo-R^2^ = 0.412. The probability calculated from threshold coefficients showed that the most likely age category for values of pulp cavity/tooth width ratio of below 0.22 is the > 60 months age class. The most likely age category for values between 0.22 and 0.32 is the 25–60 months age class, and the most likely age category for values above 0.32 is the 6–24 months age class (Fig 11).

#### Tooth wear (measured by tooth crown height)

There was no significant relationship between wild dog age class and tooth crown height, a log odds ratio of 1.391 (95% CI = 0.097 to 3.053), P = 0.056, McFadden’s Pseudo-R^2^ = 0.163.

#### Tooth wear (measured by tooth crown width/crown height ratio)

**Fig 12:**
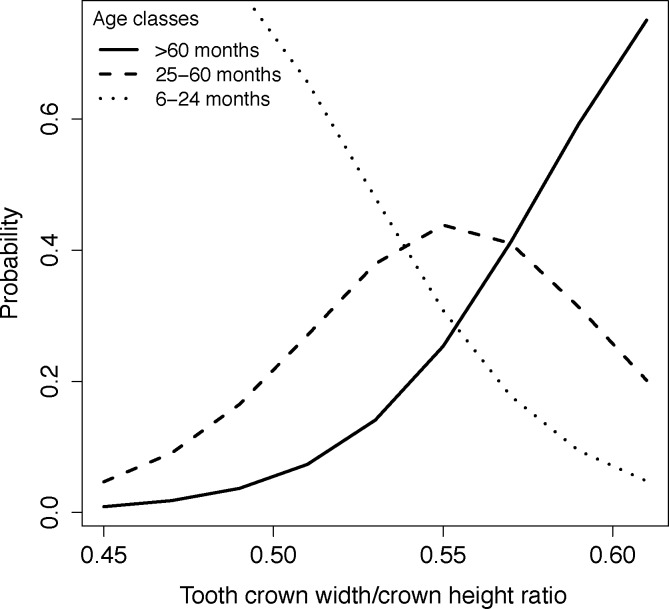
Age class and tooth crown width/crown height ratio. Plot showing the probability of each African wild dog age class (6–24 months, 25–60 months and > 60 months) in relation to tooth wear (measured by tooth crown width/crown height ratio) of African wild dog left maxillary canines.

A unit increase in tooth crown width/crown height ratio was significantly associated with a decrease in the log odds of being in the 6–24 months age class, with a log odds ratio of -36.38 (95% CI = -79.069 to -4.545), P = 0.047, McFadden’s Pseudo-R^2^ = 0.187. The probability calculated from threshold coefficients showed that the most likely age category for values of tooth crown width/crown height ratio of below 0.535 is the 6–24 months age class. The most likely age category for values between 0.535 and 0.570 is the 25–60 months age class, and the most likely age category for values above 0.570 is the > 60 months age class (Fig 12).

#### Tooth weight

There was no significant relationship between wild dog age class and tooth weight, a log odds ratio of -0.326 (95% CI = -1.545 to 0.798), P = 0.569, McFadden Pseudo-R^2^ = 0.012.

#### Skull measurements

There was no significant relationship between wild dog age class and skull length, a log odds ratio of -0.050 (95% CI = -0.218 to 0.093), P = 0.503, McFadden’s Pseudo-R^2^ = 0.026. The relationship between age class and skull width was not significant, a log odds ratio of -0.065 (95% CI = -0.287 to 0.114), P = 0.496, McFadden’s Pseudo-R^2^ = 0.027. The relationship between age class and skull height was also not significant, a log odds ratio of 0.051 (95% CI = -0.188 to 0.305), P = 0.668, McFadden’s Pseudo-R^2^ = 0.010.

## Discussion

A number of methods exist to assign age and age class to individuals based on the examination of teeth. However there is a great need for a reliable age estimation method for wild dogs that employs a simple and easily applied technique. It is also important to remember that any error in age estimation may seriously bias estimates of age-dependent life-history parameters and this may affect our understanding of population dynamics and may result in flawed management strategies [29]. This study has shown that linear equations obtained from the relationship between chronological age and (i) age estimated by counting cementum annuli, (ii) pulp cavity/tooth width ratio and (iii) tooth wear (measured by tooth crown height) can be applied to estimate the chronological age of unknown age wild dogs. Age estimated by counting cementum annuli was the most reliable method for estimating chronological age of wild dogs with a 79% predictive capacity.

Numerous age determination studies across a wide range of species have reported counting cementum annuli as the most accurate age determination method. Earlier studies by Smuts, Anderson [16] found a significant relationship between cementum lines and known age in lions (*P*. *leo)* with a very high predictive capacity of 97%. Boertje, Ellis [25] studied the accuracy of moose (*Alces alces)* age determination from cementum annuli and found a significant relationship between known age and number of cementum annuli, with 74% of 76 canines being assigned a correct age. A strong relationship was also found between number of cementum annuli lines and age of polar bears (*Ursus maritimus)*, with the most experienced cementum annuli reader scoring 75% of the specimens to their correct known age [30].

Pulp cavity/tooth width ratio and tooth wear (measured by tooth crown height) were also reliable methods for estimating chronological age with a predictive capacity of 68% and 38% respectively. Pulp cavity ratios from radiographed canines have been found to be a reliable measure of the age of canids such as dingoes (*C*.*l*. *dingo)*, with 91% accuracy for animals between 8 and 117 months [31]. Furthermore tooth wear has been used as a non-invasive method of estimating age in a range of species from white tailed deer (*Odocoileus virginianus)* [32] to gray wolves (*C*. *lupus)* [19]. Klein, Wolf [33] found a strong negative relationship between crown height and age in red deer (*Cervus elaphus)*.

Within the taxonomic rank, order, the type of teeth used to measure tooth wear varies. For artiodactyls tooth wear is assessed on the mandibular or maxillary premolar and molar teeth [13, 34]. Whereas for most carnivorans, age determination by tooth wear has been analysed by measuring the erosion of incisor and canine teeth. However, Schaller [35] measured the relative amount of wear on P^3^ teeth in lions (*P*. *leo*) to assign skulls to five age classes (sub-adult, young adult, prime, past prime and old). When using tooth wear to determine age it is also important to remember that there is a variation in tooth wear between free ranging and captive bred individuals of the same species [36, 37]. In the rhesus macaques (*Macaca mulatta*), the effects of nutrition on dental development resulted in the average eruption time of the deciduous teeth accelerating by two weeks in captive individuals compared to wild individuals, and this was attributed to improved nutrition for captive individuals [34].

The distribution of data points in the regression plots of pulp cavity/tooth width ratio and of tooth crown height suggested that assignment of age becomes less reliable in older individuals, with age estimation becoming less consistent after the age of 60 months. When wild dogs over 60 months old were removed from the analysis, the predictive capacity of the linear regression model for the relationship between pulp cavity/tooth width ratio and chronological age and the relationship between tooth wear (measured by tooth crown height) and chronological age increased from 68% to 74% and from 38% to 60% respectively. Smuts, Anderson [16] found that closure of the pulp chamber in lions (*P*. *leo)* appeared to cease at about eight to nine years meaning that the pulp chamber never becomes entirely closed resulting in no further decrease in the pulp chamber width with age, thus giving an exponential relationship. Harris, Cresswell [38] and Delahay, Walker [39] also found the relationship between age and tooth wear in European badgers (*Meles meles)* to be exponential because badger teeth wear flat in a short period of time relative to their potential life span, hence it becomes difficult to differentiate between age classes in older badgers.

Counting cementum annuli was the most reliable methods for separating wild dogs into the three age classes (6–24 months, 25–60 months and > 60 months age), with a predictive capacity of 0.705 (McFadden's Pseudo-R^2^). Poole, Lee [20] were able to use cementum annuli counts to separate between juvenile and adult wolverines (*Gulo gulo)*. Pulp cavity/tooth width ratio was also able to reliably separate wild dogs into the three age classes, with a predictive capacity of 0.412 (McFadden's Pseudo-R^2^). The ratio of pulp cavity to tooth width has also been used to effectively separate juveniles from older age classes in a number of studies [8, 20, 21, 40, 41].

This study found no relationship between tooth weight and both chronological age and age class. This could be because tooth weight is partially dependent on water content of the tooth, and factors such as the time the tooth spends in boiling water and time spent drying could affect its value. The relationship between skull measurements and both chronological age and age classes was also not significant. Nonetheless Gay and Best [42], found a significant difference in skull length and skull width between puma (*Puma concolor)* age classes, growth of the skulls continued throughout most of the animal's life until about 7–9 years for males and 5–6 years for females. Smuts, Anderson [16] also found that lion (*P*. *leo)* skull length, skull width, mandible length and mandible height increased with age and they concluded that growth curves and equations from these relationships could be reliably used to estimate age from unknown age skulls if the sex is known. However in our study the interaction between skull measurements and sex was not significant, meaning that sex had no effect on the relationship between wild dog age and skull measurements. Some studies have found that the relationship between skull measurements and age is more accurate for young individuals [16]. Nonetheless, in this study we could not test this, as we did not have enough data to run any meaningful analysis with just young individuals.

The goal of our study was to enable researchers and fieldworkers to (i) predict the chronological age and age class of deceased wild dogs using skulls and teeth measurements and (ii) predict the chronological age and age class of living wild dogs during immobilization, using teeth measurements. Although counting cementum annuli is the most reliable age determination method, it requires tooth extraction and preparation of microscopic sections and is therefore destructive, time consuming and expensive. As a result this method cannot be used in living individuals and in cases where it is not acceptable to extract teeth for welfare, ethical, religious, cultural, or scientific reasons and this complicates its use under field conditions [43, 44].

Pulp cavity/tooth width ratio is the second most reliable method for estimating chronological age and age class; this method previously needed the dog or tooth to be transported to a facility capable of taking radiographs. However, current advances in technology have brought about portable handheld dental x-ray units making it possible to take radiographs in the field on both living and dead animals. This method nonetheless requires teeth without a complicated crown fracture because at the time of pulp exposure the tooth will undergo inflammation that invariably will end in pulp death. A living pulp is required to deposit secondary dentine throughout the life of the tooth in order to be able to use pulp width changes as a criterion. Pulp cavity/tooth width ratio is a potentially relatively rapid, inexpensive and reliable method for estimating the chronological age and age class of wild dogs, both living and deceased.

Tooth wear (measured by tooth crown height) is the third most reliable method for estimating chronological age and it is the most cost effective and most convenient to measure in the field on both living and dead animals and also places a minimum stress on living animals [9]. However, estimating the age of animals using tooth wear equations from other studies may be subject to error as a result of variations between individuals, diets, habitats and populations [15, 39, 45]. Although the performance of pulp cavity/tooth width ratio and tooth wear was not as precise as counting cementum annuli, these two methods could be used in cases where precision is not paramount.

Model selection showed that a combination of counting cementum annuli and tooth crown height was the best model to explain chronological age, with a predictive capacity of 88%. Hence where possible and resources allowing, both these measurements can be taken to get the best estimate of chronological age. We removed the variable counting cementum annuli from the model selection to see if a combination of the variables measurable in the field (pulp cavity/tooth width ratio and tooth wear), can give a better prediction of chronological age. Nevertheless, the model with pulp cavity/tooth width ratio was the best model with AICc weight (0.841) while the combination of pulp cavity/tooth width ratio and tooth wear did not improve the predictive capacity of the model. Therefore the use of pulp cavity/tooth width ratio by itself is still the next best method for estimating chronological age in the absence of counting cementum annuli method.

## Conclusions

An ideal age estimation method should be accurate, the data should be easy to collect in the field, and should be cost effective. This study recommends the use of cementum annuli counts to estimate both the chronological age and age class of wild dogs whenever tooth extraction is possible. Nonetheless pulp cavity/tooth width ratio is the next best method for wild dog age estimation that gives a balance between accuracy, cost and practicability. Our study also shows a reasonable result for the relationship between wild dog chronological age and tooth wear (measured by tooth crown height), and this justifies the use of the technique in the absence of more refined methods. This paper provides the best available equation for estimating wild dog age based on pulp cavity/tooth width ratio (log_2_ (y) = 1.445–1.679 log_2_ (x); R^2^ = 0.682; 95% CI = -2.432 to -0.925; y: wild dog chronological age; x: pulp cavity/tooth width ratio), and this is probably the most practical age determination method in the field.

## Supporting Information

S1 Fig Raw data for the manuscript. This is the raw data used in all the analysis in this manuscript.
(PDF)
