# Supplementary material for: Evaluation of the Applicability of Different Age Determination Methods for Estimating Age of the Endangered African Wild Dog (Lycaon Pictus)
Source: PLoS One. 2016 Oct 12;11(10):e0164676. doi: 10.1371/journal.pone.0164676 (PMC5061369; doi:10.1371/journal.pone.0164676)
Supplement: S1 Fig — This is the raw data used in all the analysis in this manuscript. (PDF) [file pone.0164676.s001.pdf]

ID	Sex	Age class	Chronological age (months)	Cementum Age (months)	Skull length (mm)
DWD001	?		?	12	215
DWD003	?	0-6 months	1.5	0	-
DWD005	Female		?	84	199
DWD007	?		?	0	205
DWD008	Male	6-24 months	9	24	218
DWD009	Male		?	24	217
DWD013	Male		?	60	215
DWD016	Female	6-24 months	9.5	0	192
DWD017	Male		?	36	214
DWD018	Female		?	84	204
DWD019	Male	6-24 months	11	12	217
DWD021	Male	6-24 months	13	36	206
DWD023	?		?	24	209
DWD030	Female	25-60 months	28	24	196
DWD031	Male	0-6 months	2.5	0	-
DWD032	Male		?	60	224
DWD034	?		?	12	205
DWD035	Male	25-60 months	35	36	208
DWD036	Male		?	24	217
DWD037	Female		?	24	213
DWD038	Male	>60 months	80	132	219
DWD039	Female	6-24 months	14	24	203
DWD040	?		?	60	215
DWD042	Female	>60 months	90	72	212
DWD056	Male	6-24 months	19	12	-
DWD057	Male	25-60 months	60	60	-
DWD058	Female	25-60 months	48	48	-
DWD059	Male	>60 months	108	132	-
DWD060	Female	25-60 months	60	48	-
DWD061	Female	6-24 months	24	12	-

Skull width (mm)	Skull height (mm)	Pulp cavity/tooth width ratio	Crown height (mm)	Crown width/crown height ratio	Tooth weight (g)
128	100	0.482	6.010	0.517	4.036
-	-	-	-	-	-
124	93	0.272	5.680	0.472	3.679
118	94	-	-	-	-
132	102	-	6.120	0.520	4.804
132	102	0.302	6.120	0.520	4.804
137	106	0.228	5.800	0.538	5.388
112	90	0.654	5.370	0.507	2.317
137	115	0.314	4.500	0.538	5.197
128	99	0.174	5.330	0.529	3.870
125	108	0.542	6.210	0.499	4.273
131	99	0.336	5.560	0.572	4.857
129	98	0.294	5.460	0.493	3.652
117	92	0.278	4.910	0.483	2.506
-	-	-	-	-	-
143	117	0.391	5.850	0.525	5.289
129	107	0.348	5.420	0.509	3.488
130	101	0.331	5.820	0.540	4.666
128	108	0.279	5.780	0.516	4.468
131	100	0.319	5.280	0.517	3.691
137	102	0.150	5.210	0.601	5.604
128	104	0.428	5.500	0.527	3.233
139	102	0.350	5.820	0.555	5.226
126	98	0.287	4.440	0.581	3.606
-	-	0.371	4.790	0.468	4.430
-	-	-	-	-	-
-	-	0.174	3.500	0.574	4.760
-	-	0.209	3.910	0.540	4.110
-	-	0.243	3.910	0.547	3.860
-	-	-	-	-	-
